# Neonatal Feeding Trajectories in Mothers With Bipolar Disorder Taking Lithium: Pharmacokinetic Data

**DOI:** 10.3389/fphar.2021.752022

**Published:** 2021-09-22

**Authors:** Maria Luisa Imaz, Klaus Langohr, Mercè Torra, Dolors Soy, Luisa García-Esteve, Rocio Martin-Santos

**Affiliations:** ^1^ Perinatal Mental Health Clinic-BCN Unit, Department of Psychiatry and Psychology, Hospital Clínic, Centro de Investigación Biomédica en Red en Salud Mental (CIBERSAM), Institut D’Investigacions Biomèdiques August Pi I Sunyer (IDIBAPS), University of Barcelona (UB), Barcelona, Spain; ^2^ Department of Medicine, Institute of Neuroscience, University of Barcelona (UB), Barcelona, Spain; ^3^ Departament of Statistics and Operations Research, Universitat Politècnica de Catalunya, Barcelona, Spain; ^4^ Pharmacology and Toxicology Laboratory, Biochemistry and Molecular Genetics Service, Biomedical Diagnostic Center (CBD), Hospital Clínic, IDIBAPS, and Department of Medicine, UB, Barcelona, Spain; ^5^ Division of Medicine, Pharmacy Service, Hospital Clínic, IDIBAPS, UB, Barcelona, Spain

**Keywords:** bipolar disorder, lithium, breastfeeding, exclusive maternal breastfeeding, mixed breastfeeding, formula feeding, pharmacokinetics, mother-infant dyad

## Abstract

**Purpose:** Women who take lithium during pregnancy and continue after delivery may choose to breastfeed, formula feed, or mix these options. The aim of the study was to evaluate the neonatal lithium serum concentrations based on these three feeding trajectories.

**Methods:** We followed 24 women with bipolar disorder treated with lithium monotherapy during late pregnancy and postpartum (8 per trajectory). Lithium serum concentrations were determined by an AVL 9180 electrolyte analyser with a 0.10 mEq/L detection limit and a 0.20 mEq/L limit of quantification (LoQ).

**Results:** There was complete lithium placental passage at delivery, with a mean ratio of lithium concentration in the umbilical cord to maternal serum of 1.12 ± 0.17. The median times to LoQ were 6–8, 7–8, and 53–60 days for formula, mixed, and exclusive breastfeeding respectively. The generalized log-rank testing indicated that the median times to LoQ differ according to feeding trajectory (*p* = 0.037). According to the multivariate analysis-adjusted lithium serum concentrations at birth, times to LoQ are, on average, longer under exclusive breastfeeding (formula, *p* = 0.015; mixed, *p* = 0.012). No lithium accumulation was observed in infants under either exclusive or mixed breastfeeding. During the lactation follow-up, there was no acute growth or developmental delays in any neonate or infant. Indeed, lithium concentrations in the three trajectories declined in all cases. However, the time needed to reach the LoQ was much longer for those breastfeeding exclusively.

**Conclusions:** In breastfeed infant no sustained accumulation of lithium and no adverse effects on development or growth were observed.

## Introduction

Breastfeeding is an important public health issue because it promotes health, prevents disease, and contributes to reducing health inequalities in mothers and nursing infants (US Surgeon, 2011). Human milk is tailored to meet the nutritional needs of human newborns and infants, including those who are premature and sick; it provides an optimal balance of nutrients in an easily digestible and bioavailable form (James et al., 2009). Ideally, breastfeeding should be used for the first 6 months of life where possible, followed by a combination of breast milk with appropriate complementary foods until at least 1–2 years (WHO/UNICEF, 2003; AAP, 2012). For this purpose, exclusive breastfeeding is defined as the baby receiving breast milk, with the possible inclusion of vitamins or minerals through drops and syrups (Labbock et al., 2012). Exclusive breastfeeding is the reference model against which all alternative feeding methods should be measured regarding growth, health, development, and other short- and long-term outcomes. However, a common reason for not starting or for interrupting breastfeeding is medication transfer and risk of infant toxicity. Evidence-based perinatal psychopharmacology is driven by the need to balance the disease-related risks (i.e., the natural course of the bipolar disorder) and any risks to the mother, fetus or infant related to the exposure to medication.

Lithium is an effective first-line treatment for bipolar disorder (Yatham et al., 2018). Women who discontinue treatment during the perinatal period are at high risk of relapse (Vigera et al., 2007). Continued lithium prophylaxis during pregnancy may not only maintain mood stability during pregnancy but also prevent postpartum relapse (Poels et al., 2018). Lithium is a monovalent cation that is absorbed rapidly after oral intake, and it is not metabolized or bound to proteins, being eliminated almost exclusively *via* the kidneys (Granjean and Aubry, 2009). Anatomic and physiological changes during pregnancy may progressively alter the pharmacokinetics of lithium over the course of the three trimesters (Feghali et al., 2015; Westin et al., 2017; Allegaert, 2020; ). In the third trimester, lithium clearance has been found to rise by 30–50%, which may require an adjustment of the dose guided by therapeutical drug monitoring (Granjean and Aubry, 2009; Wesseloo et al., 2017). Lithium has complete placental passage, with an ion equilibration across placental barrier that is remarkably uniform across a wide range of maternal concentrations (0.2–2.6 mEq/L) (Newport et al., 2005). Due to the very low molecular weight and lack of protein binding, lithium is readily transferred to breastmilk (Hale and Rowe, 2017). Lithium excreted in human breast milk is highly variable, being approximately 50% (range 0.17–1.07%) of the mother serum concentration (Imaz et al., 2019; Newmark et al., 2019). The amount of lithium that receives the infant depends on several factors, such as the volume of milk transfer to the infant, the concentration of the lithium in the milk, and the infant’s ability to absorb (Lawrence, 1994). On the other hand, lithium is excreted almost entirely by the kidneys and is freely filtered by the glomeruli. Fractional excretion of lithium is 20%, and 60% of the filtered lithium is reabsorbed in the proximal tubule and 20% in the loop of Henle and the collecting duct (Zhuo and Li, 2013). The tubular secretion is immature at birth and approaches adult values by 7–12 months of age (Zhang et al., 2019). However, the glomerular filtration rate matures more rapidly than tubular secretion, resulting in a glomerutubular imbalance in neonates.

Lithium use while breastfeeding is a controversial topic, due to the potential risk to the neonate of lithium accumulation and secondary toxicity, especially among those who are preterm and sick (Malhi et al., 2017; Galbally et al., 2018). Two systematic reviews of clinical studies into lithium use during breastfeeding found limited evidence about whether one should initiate, maintain, or discontinue lithium during breastfeeding (Imaz et al., 2019; Newmark et al., 2019).

At present, a woman who takes lithium during pregnancy and continues after delivery may choose either breastfeeding or formula feed, or may combine these options. Given the paucity of clinical data on side effects in the infant, pharmacokinetic data may help to assess the safety of breast milk. Infant exposure to lithium is most accurately determined by measuring the drug concentration in an infant’s serum (FDA, 2005).

In this study, we hypothesized that lithium would not be accumulated in infants under either exclusive or partial breastfeeding. Our aim was to evaluate neonatal lithium concentrations in different feeding trajectories to help clinicians and patients make informed decisions about lithium use and breastfeeding during lactation.

## Methods

### Subjects and Assessments

We included data from 24 women with bipolar disorder who received lithium monotherapy and were clinically stable in late pregnancy in our university hospital between 2006 and 2018. Among these, eight women each were included into three groups, as defined by the World Health Organization (WHO, 2008): exclusive breastfeeding, mixed breastfeeding (i.e., 50–80% breastfeeding) or formula feeding. The participants were required to meet the DSM-IV-R or DSM-V criteria for bipolar I, bipolar II, or bipolar NOS. We also ensured that they were not taking concomitant medication that could interact with lithium (e.g., ibuprofen). The study was approved by the Ethics Committee for Drugs Research of the Institution (CEIm: HCD/2020/1305).

At a prenatal visit (33–35 gestational weeks), consistent with current best practice, all women were informed of the risks and benefits of lithium use during lactation. The patient and psychiatrist then collaborated to write a birth and breastfeeding plan that included clinical management, lithium monitoring in the mother and infant, and strategies to minimise postpartum sleep disruption (i.e., partner/parent support overnight for infant care) (Doan et al., 2014). All women were taking lithium twice a day to maintain constant serum lithium concentrations (Malhi and Tanious, 2011). Women were advised to suspend lithium administration at the onset of labour in the event of spontaneous deliveries, or 12 h before a scheduled caesarean section or induction. Lithium was then restarted at the same dose 6 h after vaginal delivery and 12 h after caesarean section. During subsequent follow-up visits, a senior psychiatrist evaluated the psychopathological state of the mothers, and adjusted the dose of lithium if necessary.

The neonate underwent standard follow-up. At 12–24 h of life, a neonatologist performed a systematic physical examination; at 48 h, a paediatric nurse performed a screening assessment; and, after hospital discharge, infants were evaluated regularly by a paediatrician (e.g., for weight, length, cranial circumference, neurodevelopment, and vaccination schedule) according to the standard clinical protocol of the Public Health Agency of Catalonia (ASPCAT, 2019).

### Blood Sample Collection

At delivery, we collected 10 ml paired samples of cord blood and maternal venous blood. During lactation, two paediatric nurse phlebotomists simultaneously collected venous blood from mothers (5 ml) and infants (2 ml), at 10–11 am, before the mother took her first daily dose of lithium. To study the behaviour of serum lithium concentrations during the lactation period we tried to collect samples on days 2, 7 ± 2, 15 ± 2, 30 ± 5, and 60 ± 5 postpartum; however, this was not always possible due to the technical difficulty involved and the postpartum schedules, and so the time intervals were irregular. There were no adverse incidents in the mothers or neonates/infants during blood extraction. We stopped neonatal/infant blood analysis at the request of the mother, and if serum lithium concentrations were below the limit of quantification (LoQ) of 0.20 mEq/L on two consecutive occasions. The sample at 48 h postpartum was obtained during the newborn screening assessment.

### Serum Lithium Analysis

For lithium analysis, we collected maternal, cord, and neonatal/infant venous blood samples in BD Vacutainer® no-additive Z plus tubes (BD Diagnostics, Preanalytical Systems, Franklin Lakes, NJ07417). After allowing the blood to clot in an upright position for at least 30 min, serum was separated by centrifugation at approximately 3,000 rpm for 10 min and analysed as soon as possible. If storage was required, the serum samples were capped and refrigerated at 4°C–8°C until analysis. Lithium concentrations were determined by an AVL 9180 electrolyte analyser based on the ion-selective electrode measurement principle (Roche Diagnostics 9,115 Hague Road Indianapolis, IN46256). Two-point calibration was performed every 4 h with a measurement range between 0.1 and 6 mEq/L. The limit of detection was 0.10 mEq/L and the LoQ was 0.20 mEq/L. Expressed as a percentage (coefficient of variation), the within-day precision was 0.97–4.1% and the between-day precision was 1.3–6.4%.

### Lithium Concentration Analysis

The ratio of lithium concentration in the umbilical cord to that in the maternal serum was calculated for each maternal-infant pair as the lithium placental passage index. The infant serum lithium concentration was monitored from birth until the time when the serum lithium concentration reached the LoQ or below. Since lithium could be taken at arbitrary times, the time of interest was interval-censored between the last day that the lithium level was above the LoQ and the first day that it was equal to or below the LoQ.

### Statistical Analysis

All data were analysed using SPSS for Windows (Version 25; IBM Corp., Armonk, NY, United States) and the statistical software package R (V4.0.2; The R Foundation for Statistical Computing, Vienna, Austria). A descriptive analysis was performed to characterize the sample and the placental passage of lithium, using either absolute and relative frequencies or means, standard deviations, and ranges as appropriate.

For the analysis of the interval-censored data, we used the Turnbull estimator to estimate the probability that the LoQ was reached as a function of time (Turnbull, 1976). The generalized log-rank test for interval-censored data based on a vector distribution was used to test whether the median times until the LoQ differ among feeding trajectories (Fay and Shaw, 2010; Oller and Langohr, 2017).

To adjust the comparison of the feeding trajectories for the lithium concentrations at birth, the Weilbull regression model was applied. This parametric model models the logarithm of the times of interest assuming that these times follow a Weibull distribution (Gómez et al., 2009). Following, post-hoc comparisons were carried out to compare the feeding trajectories in the frame of this model and the corresponding confidence intervals and *p*-values were adjusted for multiple comparisons.

## Results

### Characteristics of the Samples

Most participants were Caucasian (96%; *n* = 23), the mean (±SD) age was 33 ± 3.8 years, around half (54%; *n* = 13) had university level education, and all were married or had a partner. Obstetrically, 19 (80%) of the mothers were primiparous and 9 (37.5%) had deliveries by caesarean section. The three study groups (exclusive breastfeeding, mixed breastfeeding, and formula feeding) were similar with respect to sociodemographic and obstetric characteristics.

Two women relapsed postpartum. One woman in the exclusive breastfeeding group needed a brief hospitalization at day 45 postpartum for a manic episode with psychotic features despite having a therapeutic lithium concentration (0.91 mEq/L). Another woman in the mixed breastfeeding group had a manic relapse at 36 days, but with subtherapeutic serum levels (0.21 mEq/L), and her levels improved with outpatient therapy. Breastfeeding was stopped in both patients.

All neonates were full-term newborns (37.4–41.2 weeks) and had an adequate weight for gestional age, mean ± SD (range) 3,478 ± 455 g (2,500–4,400 g). Table 1 shows the gender and lithium concentrations at delivery for the 24 full-term neonates by their feeding trajectory. As shown, the three study groups were similar with respect to gender and the umbilical cord and maternal lithium concentrations. There was complete lithium placental passage at delivery: the mean ratio of lithium concentration between the umbilical cord and maternal serum was 1.12 ± 0.17. Although most neonates at delivery did not show signs of lithium toxicity or other adverse clinical events, adverse effects were recorded in six (25%). In the exclusive breastfeeding group, there were two cases (25%) of transient hypotonia and one case (12.5%) of isolated low-set ears. In the mixed breastfeeding group, three (37.5%) had respiratory distress following operative delivery (i.e., forceps or caesarean): one needed neonatal resuscitation in the delivery room (cord lithium, 0.95 mEq/L) and two required admission to the neonatal intensive care unit for 24 h (cord lithium, 0.55 and 0.96 mEq/L). In the formula feeding group, there was a single case (12.5%) of transient hypertonia.

**TABLE 1:** Descriptive statistics of neonates according to feeding trajectory.

	All *N* = 24	Exclusive *N* = 8	Mixed *N* = 8	Formula *N* = 8
**Gender**				
Female	12 (50%)	4 (50%)	5 (62.5%)	3 (37.5%)
Male	12 (50%)	4 (50%)	3 (37.5%)	5 (62.5%)
**Intrapartum serum lithium concentration^a^**				
Umbilical cord	0.48 (0.22) (0.23–0.96)	0.44 (0.16) (0.23–0.76)	0.58 (0.29) (0.26–0.96)	0.42 (0.17) (0.25–0.68)
Maternal serum	0.43 (0.20) (0.19–0.95)	0.39 (0.16) (0.19–0.72)	0.50 (0.27) (0.22–0.95)	0.40 (0.15) (0.25–0.64)
Ratio	1.12 (0.17) (0.63–1.53)	1.13 (0.11) (1.02–1.30)	1.16 (0.16) (1–1.53)	1.06 (0.22) (0.63–1.28)

Data are presented as either N (%) or Mean (SD) (range).

a mEq/L, including the ratio of umbilical cord to maternal serum levels.

The 48 h newborn screening assessment was in all cases normal. Postpartum neonatal thyroid function (TSH) was between the normal ranges (0.10–5.65 mU/mL) in all cases. Neonatal bilirubinemia results (by transcutaneous bilirubinometers) were below 12 mg/dl (2.50–11.50 mg/dl) in all cases. The physiological weight loss in the first 48 h was 9.3% (8.86–9.76%).

The mean ± SD (range) hospitalisation period was 2.83 ± 0.86 days (2–4 days).

Finally, paediatricians observed no growth or developmental delays in any of the infants during follow-up.

### Lithium Concentrations

We collected a total of 138 lithium serum samples, 24 samples from mother-infant pairs at delivery, and 90 samples from neonates during lactation.

**FIGURE 1:**
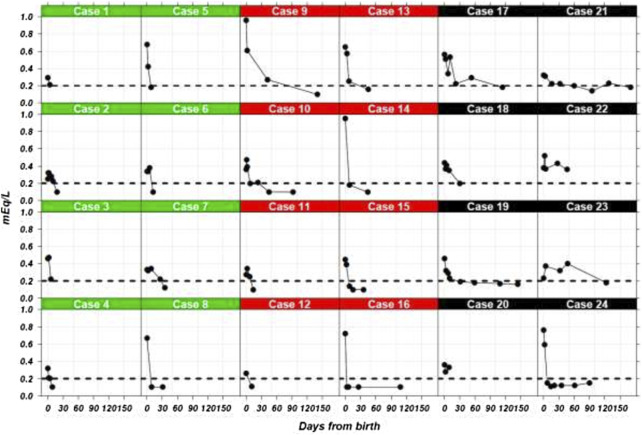
Lithium serum concentrations in 24 neonates from birth. Three groups of neonates are shown by type of feeding. Cases 1–8 (green), 9–16 (red), and 17–24 (black) show the results formula-fed, mixed-fed, and exclusive breastfed neonates, respectively. The dotted line indicates the 0.20 mEq/L limit of quantification.

Figure 1 shows the time course data of the serum lithium concentrations for each of the 24 neonates, where the dotted line represents the limit of quantification (LoQ) of 0.20 mEq/L. However, seven cases [fourth in formula group (cases 2, 3, 6 and 7), two in the mixed breastfeeding group (cases 10 and 11), and one case in the exclusive breastfeeding group (case 22)] showed a transient increase of serum lithium concentration (<0.15 mEq/L; range: 0.01–0.14) probably associated to the physiological weight loss. In the exclusive breastfeeding group, one case (case 23) showed also a transient increase (0.14 mEq/L) because of physiological weight loss and drastic increase in maternal level of lithium concentration (0.19 mEq/L to 1.09 mEq/L); and another (case 17) showed an increase of 0.19 mEq/L on the 11th day of life because the infant’s serum sample was hemolyzed.

Supplementary Figure S1 presents the interval-censored times until the time when lithium levels fell below the LoQ; the exact moment was not observed and was only known to lie in these intervals. After 40 days, the lithium serum concentration of only 4 neonates with exclusive breastfeeding was definitely below the LoQ, compared to 7 neonates with mixed feeding and 6 with formula feeding.

### Univariate Nonparametric Analysis

**FIGURE 2:**
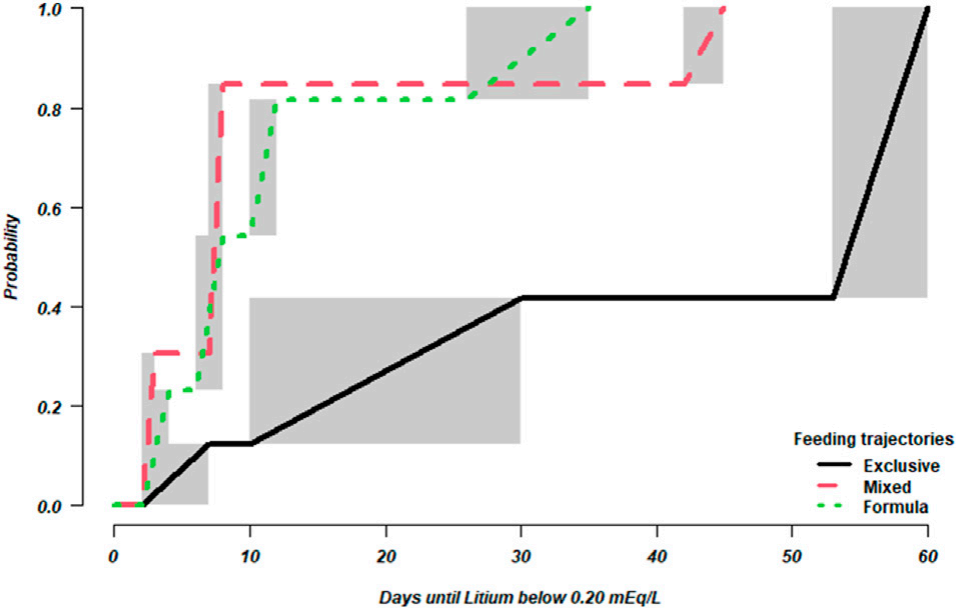
Estimated cummulative probability that lithium serum concentrations falls below LoQ. Shaded areas indicate that the estimation of the probability is not defined in the corresponding intervals, but only known to increase monotonically.

Figure 2 (and Supplementary Table 1) shows the estimated probabilities that the lithium levels falls below the LoQ as a function of time from birth according to the Turnbull estimator. The median times until LoQ lay between 6–8 days (formula feeding), 7–8 days (mixed), and 53–60 days (exclusive breastfeeding). According to the results of the generalized log-rank test to compare the lactation types (χ2 = 6.8, df = 2, *p* = 0.037) times to LoQ differed between the feeding trajectories.

### Multivariate Analysis

Table 2 provides the parameter estimates of the Weibull regression model, which was used to compare the feeding trajectories while adjusting for the lithium concentrations at birth, and shows the results of the pairwise post-hoc comparisons. The differences observed among breastfeeding trajectories are not explained by different lithium levels at birth and we can therefore state that, on average, the times to LoQ were longest under exclusive breastfeeding; no statistically significant differences were found between the mixed and the formula trajectory.

**TABLE 2:** Parameter estimates of the Weibull regression model for time until lithium serum concentrations falls below LoQ and pairwise post-hoc comparisons of feeding trajectories.

Weibull regression model				
—	Value	SE	Z	p
(Intercept)	3.566	0.582	6.126	0.000
Lithium concentration^a^ at birth	0.851	1.117	0.762	0.446
Mixed *vs* exclusive breastfeeding	−1.593	0.637	−2.501	0.012
Formula *vs* exclusive breastfeeding	−1.357	0.555	−2.444	0.015
**Pairwise comparisons**				
—	**Differences**	**Lower 95%CI**	**Upper 95%CI**	**p**
Mixed *vs* exclusive breastfeeding	−1.593^b^	−3.085	−0.102	0.033
Formula *vs* exclusive breastfeeding	−1.357^b^	−2.656	−0.057	0.038
Formula *vs* mixed breastfeeding	0.237	−1.284	1.757	0.929

Abbreviations: CI, confidence interval; SE, standard error

a mEq/L.

b The negative sign indicates larger times until lithium serum concentrations falls below LoQ under exclusive breastfeeding.

## Discussion

As far as we know, this is the first study to compare serum lithium concentrations during three feeding trajectories after delivery: namely, exclusive breastfeeding, formula feeding, and a mixed approach. Notably, no lithium sustained accumulation was found in infants under either exclusive or mixed breastfeeding. As expected, however, we found significant differences in the time taken to reach or exceed the LoQ between the feeding trajectories. All in all, the results provide substantial support for recommending maternal breastfeeding in women with lithium-responsive bipolar disorder in whom lithium prophylaxis helps to prevent postpartum affective relapse.

In a preliminary study of exclusive breastfeeding in the neonatal period, we found that lithium clearance in nursing infants was independent of maternal lithium levels, and that infant serum lithium concentrations fell over time from delivery to the third month postpartum, by 43.80% (95%CI: −38.45% to −43.88%) in the first month and by 58.52% (95%CI: −38.22% to −78.90%) at 3 months (Imaz et al., 2021). In women who had taken lithium weeks before delivery, it was shown that infant serum concentrations in the first week postpartum may reflect transplacental passage rather than intake *via* breast milk (Hale and Rowe, 2017). In the present study, we compared the pharmacokinetics of serum lithium concentrations from delivery during three feeding trajectories. The analysis of the formula feeding trajectory (in which the last infant exposure to lithium was at delivery) provided us with a reference point with which to compare the course of the exclusive and mixed breastfeeding trajectories in the first 7–10 days postpartum. Although serum lithium concentrations declined in all three trajectories, the time needed to reach the LoQ was longest in the exclusive breastfeeding trajectory. The non accumulation of lithium in breastfeeding infant could be explained by 1) the decrease of transport of lithium into the milk with age; and 2) because the neonatal lithium renal excretion increases with maturation of renal tubular transport, even though tubular function matures more slowly than glomerular function after birth (Zhang et al., 2019).

Previous systematic reviews have failed to address clinical symptoms of lithium toxicity in infants for levels <0.30 mEq/L (Pacchiarotti et al., 2016; Imaz et al., 2019; Newmark et al., 2019). We decided to stop monitoring lithium concentrations in infants when levels reached ≤0.20 mEq/L in two consecutive determinations (i.e., the LoQ) (Armbruster and Pry, 2008). We chose the time to reach the LoQ as our outcome variable because the three groups had different lithium exposures: the last exposure was at delivery in the formula group, but it continued in different proportions in the mixed and exclusive breastfeeding groups. However, neonates in all groups shared the problems of an immature renal system and the physiological loss of fluids, which may affect lithium concentrations.

With regard to the effects of transplacental lithium exposure at birth, six neonates in this sample suffered mild and transient complications that resolved before hospital discharge. During the follow-up period, paediatricians found no observable growth or developmental delay in neonates or infants in any of the three trajectories.

The postpartum period is associated with the highest lifetime risk of hospitalisation for women with bipolar disorder (Munk-Olsen et al., 2009). Lithium has proven to be an effective preventive treatment during the postpartum period (Berking et al., 2015). A systematic review and meta-analysis showed a relapse rate during postpartum in women with bipolar disorder of 37% (Wesseloo et al., 2016). In the first month postpartum, the recommended therapeutical range is ≥0.80 mEq/L (Wesseloo et al., 2017). In our sample of bipolar disorder patients under lithium monotherapy during lactation, only two patients out of 24 (fewer than 10%) relapsed during follow-up (at 36 and 45 days postpartum respectively): one with a subtherapeutic serum lithium concentration, and the other within the therapeutic range.

The study has several strengths and limitations. The strengths include its assessment of the three breastfeeding trajectories from delivery in groups with similar obstetrical and demographic characteristics. As for to limitations, the study was performed in a selected sample of bipolar women receiving lithium monotherapy during pregnancy and lactation, all of whom had full-term newborns. As such, our findings may be not generalizable to more heterogeneous populations (i.e. pre-term neonates; sick infants; lithium in polytherapy, and so on). The amount of lithium transferred to breastfed infant could be measure directly in infant serum or estimated on the basis of pharmacokinetics parameters [i.e. milk to maternal plasma drug concentration ratio (M/P ratio), the relative infant dose (RID)]. The infant serum concentration provides information regarding the fraction of drug that is systematically available to the breastfed child (Begg et al., 2002). It is the most direct measure for risk assessment (FDA, 2005). We decided to use the infant serum lithium concentration as direct measure. Having to obtain a blood sample to analyze the infant serum lithium concentration may be a limitation, as this is an invasive method that may cause pain and may be rejected by some parents. Because of this, the determination of lithium in saliva has been proposed; however, from the pre-analytical point of view, obtaining saliva in infants between 0 and 2 months is especially challenging, and in addition the therapeutic range of lithium in this matrix has not been yet defined (Murru et al., 2017). We were also aware of the limit of detectability of the assay used to measure lithium concentration, especially for values below the LoQ.

## Conclusions

We conclude that bipolar women treated with lithium monotherapy during late pregnancy (with a brief peripartum discontinuation) and postpartum may continue lithium use during breastfeeding, since it is safe and does not cause infant harm or accumulation of lithium in our sample. Lithium concentrations in the three lactation trajectories (exclusive, mixed and formula) fell in all cases. However, the time needed to reach the LoQ was much longer in the case of mothers who breastfed exclusively.

Clinical follow-up is required throughout the postpartum period to ensure the safety of both the mother and infant. We recommend that infant lithium serum concentrations be monitored peripartum at 2 days and 1 week postpartum for all trajectories, with additional monitoring at 1 and 2 months postpartum for those who exclusively breastfeed. Later on, if infant lithemia is < 0.20 mEq/, lithium monitoring may only be necessary in the case of fever, unusual behaviour, increased sedation, hypotonia, dehydration, difficulty feeding, or abnormal growth or development.

Finally, more standardized and collaborative studies are needed in larger cohorts to better elucidate the extent of maternal-infant lithium transfer and the effects of breast milk exposure on infant health and development.

## Data Availability

The raw data supporting the conclusion of this article will be made available by the authors, without undue reservation.
