# Supplementary figures and images for: Neonatal Feeding Trajectories in Mothers With Bipolar Disorder Taking Lithium: Pharmacokinetic Data
Source: Front Pharmacol. 2021 Sep 22;12:752022. doi: 10.3389/fphar.2021.752022 (PMC8493120; doi:10.3389/fphar.2021.752022)

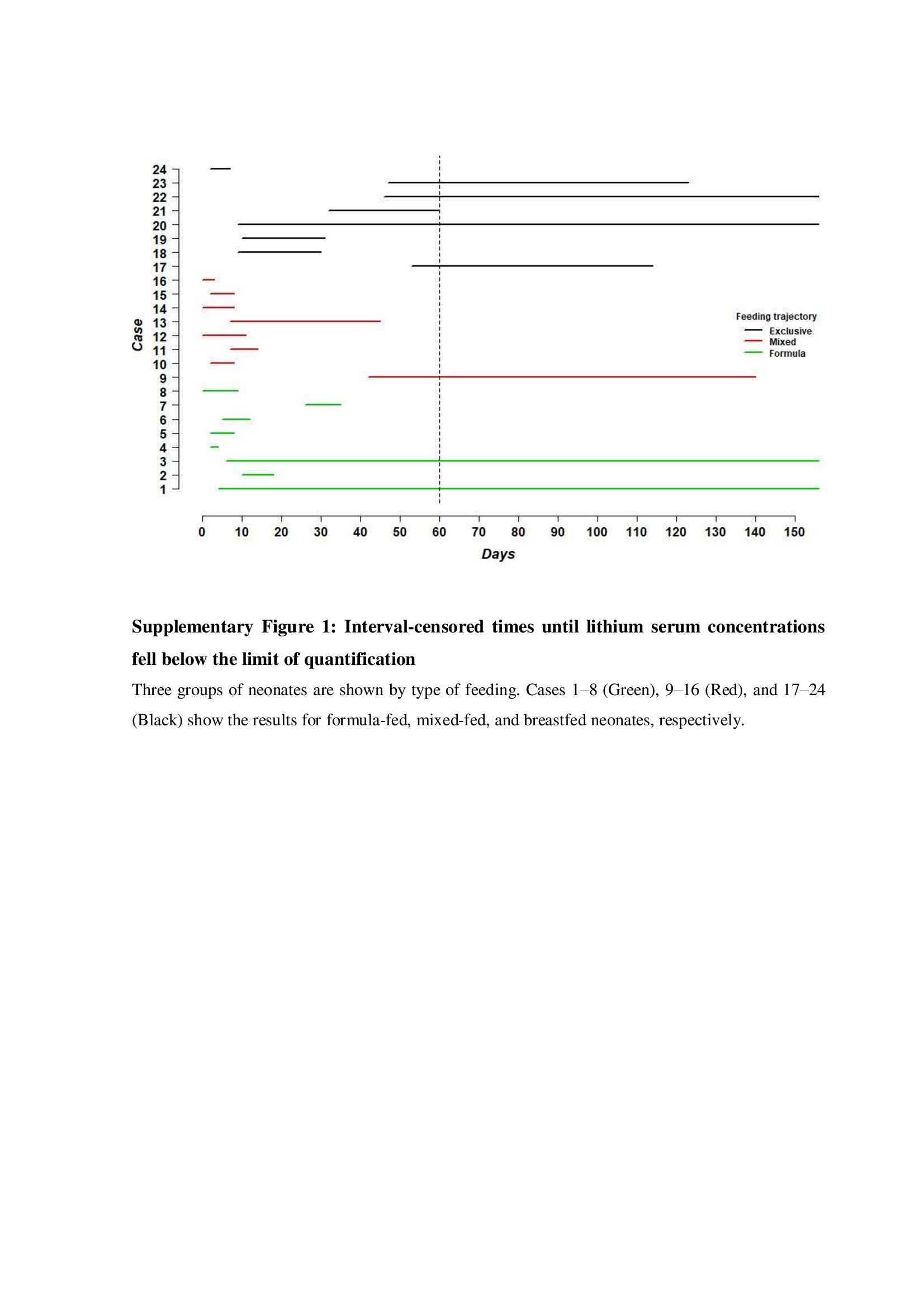

Supplement: Supplementary file 1 [file Image1.TIFF]
