# Supplementary material for: Neonatal Feeding Trajectories in Mothers With Bipolar Disorder Taking Lithium: Pharmacokinetic Data
Source: Front Pharmacol. 2021 Sep 22;12:752022. doi: 10.3389/fphar.2021.752022 (PMC8493120; doi:10.3389/fphar.2021.752022)
Supplement: Supplementary file 2 [file Table1.DOCX]

**Supplementary Table 1. Turnbull estimator for interval-censored data to estimate the probability that the limit of quantification (LoQ) is reached as a function of time.**

| **Trajectory** | **Interval (days)** | **Probability** | **Cumulative**  **probability** |
| --- | --- | --- | --- |
| **Formula feeding** (N = 8) | 2, 4 | 0.2316 | 0.2316 |
|  | 6, 8 | 0.3115 | 0.5431 |
|  | 10, 12 | 0.2716 | 0.8147 |
|  | 26, 35 | 0.1833 | 1.0000 |
| **Mixed** **breastfeeding** (N = 8) | 2, 3 | 0.3048 | 0.3048 |
|  | 7, 8 | 0.5428 | 0.8476 |
|  | 42, 45 | 0.1524 | 1.0000 |
| **Exclusive** **breast feeding** (N = 8) | 2, 7 | 0.1250 | 0.1250 |
|  | 10, 30 | 0.2917 | 0.4167 |
|  | 53, 60 | 0.5833 | 1.0000 |
